# The Role of Temperature in Determining Species' Vulnerability to Ocean Acidification: A Case Study Using *Mytilus galloprovincialis*

**DOI:** 10.1371/journal.pone.0100353

**Published:** 2014-07-01

**Authors:** Kristy J. Kroeker, Brian Gaylord, Tessa M. Hill, Jessica D. Hosfelt, Seth H. Miller, Eric Sanford

**Affiliations:** 1 Bodega Marine Laboratory, University of California Davis, Bodega Bay, California, United States of America; 2 Department of Evolution and Ecology, University of California Davis, Davis, California, United States of America; 3 Department of Geology, University of California Davis, Davis, California, United States of America; University of Hong Kong, Hong Kong

## Abstract

Ocean acidification (OA) is occurring across a backdrop of concurrent environmental changes that may in turn influence species' responses to OA. Temperature affects many fundamental biological processes and governs key reactions in the seawater carbonate system. It therefore has the potential to offset or exacerbate the effects of OA. While initial studies have examined the combined impacts of warming and OA for a narrow range of climate change scenarios, our mechanistic understanding of the interactive effects of temperature and OA remains limited. Here, we use the blue mussel, *Mytilus galloprovincialis*, as a model species to test how OA affects the growth of a calcifying invertebrate across a wide range of temperatures encompassing their thermal optimum. Mussels were exposed in the laboratory to a factorial combination of low and high *p*CO_2_ (400 and 1200 µatm CO_2_) and temperatures (12, 14, 16, 18, 20, and 24°C) for one month. Results indicate that the effects of OA on shell growth are highly dependent on temperature. Although high CO_2_ significantly reduced mussel growth at 14°C, this effect gradually lessened with successive warming to 20°C, illustrating how moderate warming can mediate the effects of OA through temperature's effects on both physiology and seawater geochemistry. Furthermore, the mussels grew thicker shells in warmer conditions independent of CO_2_ treatment. Together, these results highlight the importance of considering the physiological and geochemical interactions between temperature and carbonate chemistry when interpreting species' vulnerability to OA.

## Introduction

As carbon dioxide (CO_2_) emissions continue to rise, many organisms will experience complex changes in their environment, from shifts in temperature to altered geochemistry [1]. The combined ecological effects of multiple environmental changes can be interactive, where the presence of one environmental driver influences species' responses to a second driver [2], [3]. There is considerable concern regarding potential synergistic effects among multiple environmental changes, where the combined effect on a species of two or more drivers is worse than would be expected from a strictly additive influence of the separate factors [4]. However, research has shown that the presence of a second driver can also offset or lessen the effects of another stressor [2]. The potential for interactive effects prevents robust predictions of ecological effects of global change from the results of experiments incorporating single drivers. Understanding how organisms and ecosystems respond to key environmental drivers across simultaneous changes in other drivers remains a priority for science, management, and conservation.

Warming and ocean acidification (OA) are two of the most prominent anthropogenic changes in the ocean. Both are driven by elevated CO_2_ and threaten to have widespread ecological consequences. The magnified greenhouse effect associated with rising atmosphere CO_2_ concentrations is predicted to cause a global rise in sea surface temperatures from 2–3°C within this century [1]. In addition, the ocean absorbs a substantial proportion of the CO_2_ emitted into the atmosphere [5], causing changes in carbonate chemistry that results in reduced concentrations of carbonate ion ([CO_3_^2−^]) and a decline in seawater pH [6]. Model projections indicate that sea surface warming and OA will continue for centuries [1], [7], and research suggests these changes to the ocean could have profound consequences for marine organisms and ecosystems [8], [9]. In particular, reductions in the concentration of carbonate ion and pH associated with OA are strongly correlated with decreased calcification and growth in many marine organisms [10], [11]. On average, significant reductions in calcification (27%) and growth (11%) in response to near-future acidification scenarios are apparent despite potential species-specific variability [11]. Similarly, increased temperature is likely to cause a redistribution and local extinctions of many marine species [12].

While there has recently been considerable focus on the effects of OA on marine organisms and some attention to interactions with temperature, a general understanding of the interplay between OA and temperature remains incomplete. Several ecological experiments have tested organisms' responses to a factorial combination of OA and a moderate rise in temperature (e.g., 2–3°C), the latter based on near-future scenarios of climate change (e.g., 2100) [1]. While this research has highlighted the potential for interactive effects [11], we still lack a broader understanding of the different ways in which temperature influences species' responses to OA. For example, temperature directly affects numerous biological processes, and the effects of OA may vary due to temperature-mediated physiological differences across the wide range of temperatures that organisms naturally experience in the field. Furthermore, a point that is often neglected in experiments examining combined effects of OA and temperature is that the latter parameter also affects CO_2_ solubility in seawater and the equilibrium coefficients governing carbonate chemistry [13]. Thus, a better understanding of how warming influences species' responses to OA through both direct (e.g., warming-induced increases metabolic rates) and indirect pathways (e.g., warming-induced changes in carbonate chemistry) is necessary.

Temperature plays a fundamental role in biological processes, from biochemical reaction and metabolic rates [14], [15] to growth rates [16] and species interactions [17], [18]. Moderate warming increases biochemical and physiological rates up to a species-specific optimal temperature. Beyond this optimum, further warming can denature proteins, slow growth, and cause reductions in performance and survival [14], [19]. Although studies examining the biological effects of environmental warming have typically focused on comparisons between current conditions and those projected in the near future, coastal marine organisms regularly experience a wide range of temperatures due to temporal fluctuations (e.g., tidal and seasonal cycles) that vary geographically [20], [21]. Moreover, the changes in temperature associated with climate change will not be the same everywhere. Some places will experience more severe warming [22], while others may actually experience cooling [23]. This inherent variability in environmental temperature regimes and projections for sea surface warming underpins the importance of understanding the mechanistic links between temperature and OA, as well as how the effects of OA vary across a wide range of temperatures that species may experience across space and time in the near future.

Temperature can affect species' responses to OA through both direct and indirect mechanisms. Because temperature is fundamental to biological processes, it is likely to have a direct impact on physiological responses to OA. For example, temperature-mediated increases in metabolism and feeding rates could potentially offset reductions in growth caused by OA in food-replete conditions by increasing the scope for growth (an estimate of the net energy gained through food minus that lost through excretion and respiration) [24]–[27]. However, warming could also exacerbate reductions in growth caused by OA if the magnitude of warming is stressful for the organism [28]. In addition, warming can increase kinetics central to the process of non-biogenic [29] and biogenic calcification [30], thereby mediating reductions in calcification caused by OA. Warming could also have indirect effects on marine organisms by mediating the degree of acidification for a given atmospheric CO_2_ concentration. For example, the saturation state (Ù) of aragonite, a mineral form of calcium carbonate, is defined as the thermodynamic potential for aragonite to form or dissolve in seawater. The saturation state of aragonite (and other carbonate minerals) is greater in warmer water than colder waters at the same atmospheric CO_2_ [31]. As a consequence of such relationships, it can be difficult to distinguish direct effects of temperature on the organism from the indirect effects of temperature via changes in carbonate chemistry and calcification energetics.

For this study, we used the bay mussel *Mytilus galloprovincialis* as a model species to examine the interactive effects of OA across a range of temperatures for a calcifying invertebrate. We chose *M. galloprovincialis* because it is a eurythermal species displaying a tolerance to a wide range of temperatures (from near-freezing to ∼31°C), which is widely thought to have helped it invade many locations worldwide from its origins in the Mediterranean Sea [32]. As such, there is a wealth of knowledge regarding the thermal physiology of *M. galloprovincialis*. Physiological studies of *M. galloprovincialis* indicate its acute upper thermal tolerance (e.g., as indicated by cardiac failure) can range from 26°C to 31°C, depending on the acclimation temperature and salinity [33]. OA has also been shown to affect *M. galloprovincialis* and its congeners [24], [25], [34]–[36]. While early research suggested that OA could slow the growth of bay mussels [37], recent studies have reported high tolerance to predicted near future conditions [38]. In this experiment, we measured the growth and shell accretion of *M. galloprovincialis* (size range = 4.5–9.7 mm) in the laboratory in a factorial combination of low and high *p*CO_2_ (400 and 1200 µatm CO_2(atm)_, respectively) and a range of temperatures (12, 14, 16, 18, 20, and 24°C) over a period of one month to quantify how temperature affects their response to OA.

## Materials and Methods

### Experimental system

Carbonate chemistry was manipulated by bubbling NIST-traceable CO_2_ air mixtures at 385 µatm (hereafter referred to as our 400 µatm treatment) and 1200 µatm CO_2_ into 20 L carboys of filtered seawater (hereafter referred to as “carboy” water). During the experiment, carboys were held in separate temperature-controlled water baths at 12°C, 16°C, and 20°C. The carboys were bubbled for four days to equilibrate and reached nominal pH_T_ values of 8.06 (12°C), 8.06 (16°C), and 8.08 (20°C) for the 400 µatm CO_2_ treatment, and 7.66 (12°C), 7.67 (16°C), and 7.69 (20°C) for the 1200 µatm CO_2_ treatment. This pre-equilibrated water from the carboys was used to fill 3L insulated jars that held the mussels. Because this was a closed system, the water was replaced in the jars with the pre-equilibrated carboy water every other day. The jars were held in a water bath that was chilled to 9°C, and an individual titanium heater, temperature probe, and digital controller (True Temp T3-150, JBJ Inc.; one unit each per jar) were used to complete the temperature adjustment to one of the six prescribed experimental temperatures (±0.5°C).

The same air mixtures (400 and 1200 µatm CO_2_) were pumped into acrylic boxes mounted over the jars to maintain a headspace at the target CO_2_ concentration and thereby minimize net gas exchange across the free surface of the seawater in the jars (see [35], [39] for detailed description of experimental system). The two CO_2_ concentrations were partitioned among six different boxes, each containing six jars (one at each target temperature). Each temperature by CO_2_ combination therefore had three replicate jars. A small electric pump placed inside each box bubbled the headspace air directly into the jars to provide flow and maintain seawater chemistry in individual jars.

*Mytilus galloprovincialis* (ranging from 4.5–9.7 mm in length) were collected from the upper edge of the mussel bed dominated by *Mytilus californianus*, which defines the mid-intertidal zone at a site on the central coast of California near Santa Barbara (Mussel Shoals, CA: 34.93026°N, −120.66490°W), where they experienced daily emersion. The mean sea surface temperature for the three weeks prior to collection at MLLW at Stearn's Wharf (∼30 km from collection site) was 12.8°C±0.5°C SD (N = 8656) (Southern California Coastal Ocean Observing System; http://www.sccoos.org/), but the mussels likely experienced higher temperatures as a result of daily emersion and may have been acclimated to warmer temperatures. Although these mussels were not genotyped, locations south of Morro Bay in California are dominated by *M. galloprovincialis* [40]. *M. galloprovincialis* is an invasive species along the west coast of the United States, and permits are not required for collection. The mussels were brought to Bodega Marine Laboratory where they were kept at ∼12°C in a flow-through water system for 10 days prior to the start of the experiment, during which they received ambient food concentrations from ocean waters immediately adjacent to the marine laboratory.

At the start of the experiment, five small mussels were haphazardly assigned to each jar. Photos were taken using a camera attached to a stereomicroscope (Leica M125 with DC290 camera), and initial length was quantified from the photos using image-analysis software (Image J v1.37, NIH). Initial volume (more detailed methods below) was also estimated from the wet weight and buoyant weight (Mettler-Toledo XS204, ±0.0001 g). The five mussels were placed in a small, mesh-sided plastic cage with separate compartments so that the same individuals could be measured again at the end of the experiment. The cage was then suspended in the middle of the jar, where mussels would receive adequate flow from the bubbler.

### Acclimation

At the start of the experiment, all jars received 12°C seawater that was pre-equilibrated to one of two *p*CO_2_ levels (400 or 1200 µatm CO_2_). The temperature was then ramped 2°C every 24 hours, such that all of the jars reached the target temperatures within seven days. While changes in seawater temperature of this magnitude are likely to occur over longer time frames in the field (e.g., seasonal fluctuations occurring over months), previous research suggests acclimation/acclimatization of many of the physiological traits of *M. galloprovincialis* can occur over 3–4 weeks [33], [41] (but see [42]). The mussels were not gradually acclimated to the *p*CO_2_ treatments due to our reliance on pre-mixed gases, but it is likely that nearshore marine organisms regularly experience dramatic changes in *p*CO_2_ over relatively short time frames associated with upwelling [43] or diurnal cycling in tide pools [44].

### Experimental procedures

The water was replaced in all jars every other day with pre-equilibrated water from the carboys. Before discarding the water from the jars, it was tested for pH, DIC, and TA. In addition, the water was occasionally tested for salinity to make sure that the warming treatment was not influencing salinity. The cages holding the mussels were removed from the jars and cleaned lightly with a soft acrylic brush, while the jars were scrubbed with an acrylic pad to prevent growth of diatoms. The jars were then refilled with filtered seawater that had been pre-equilibrated to the appropriate CO_2_ and approximate temperature treatment (carboy water). During the experiment, the 12 and 14°C treatments received water from the 12°C carboys, the 16 and 18°C treatments received water from the 16°C carboys, and the 20 and 24°C treatments received water from the 20°C carboys. The heaters were then used to reach and maintain the precise target temperature in each jar. Temperature loggers in each jar indicate that the target temperatures were reached within ten minutes of replacement with new water, before mussels were returned to the jars. On average, mussels were out of the water for approximately 15–30 minutes per water change, equivalent to 1% of the experimental duration.

After a water change, the mussels in each jar were fed a combination of live microalgae (*Isochrysis galbana*) at a density of approximately 100,000 cells/ml, supplemented with a pre-determined volume of dead microalgal mixture (Shellfish Diet 1800, Reed Mariculture Inc.). In addition, each jar received a second dose of the same microalgal mixture on the subsequent day. A handheld fluorometer (Aqualflor 8000-010, Turner Designs) was used to monitor algal concentrations in the jars throughout the experiment. As the mussels grew and consumed more algae, the volume of Shellfish Diet was increased to ensure that food was available to the mussels in the warmest temperature treatment throughout the entirety of the experiment, based on the rapid clearance rates in the 24°C treatments. Mean fluorescence in the 24°C jars just before water changes was 3.2±0.3 SEM (N = 10), indicating that the lowest food concentrations prior to water changes and food replenishment were still above 20,000 cells/mL. Although the food concentration was set to accommodate the mussels in the highest temperature treatment that consumed the most food, every jar received the same amount of food. In total, we increased the daily allotment of Shellfish Diet from 200 to 700 µl in all jars from the beginning to the end of the experiment.

### Mussels' responses

Feeding rates were estimated mid-experiment (Day 19) by measuring the fluorescence of seawater samples taken from each jar directly after adding the live and dead microalgal mixture and again after approximately 20 hours of feeding. Because fluorescence is temperature dependent, the exact temperature of the seawater was noted during the fluorescence measurements and a temperature offset was used to compare fluorescence measurements taken at different temperatures (−1.4%/°C; Aqualflor 8000-010 User Manual, Turner Designs). The change in fluorescence per hour was calculated to compare feeding rates across treatments.

The length, wet weight, and buoyant weight of the mussels were re-measured after four weeks. The mussels were then dried at 80°C for 24 hours and weighed for dry weight. In addition, the mussels were put in a muffle furnace at 500°C for 8 hours and weighed for ash-free dry weight. Relative change in length was estimated by subtracting the initial length from the final length, and then dividing by the initial length. Volume was estimated by subtracting the buoyant mass from the wet mass, and then dividing by the density of seawater. Relative change in volume was estimated by dividing the change in volume by the initial volume. The ratio of soft tissue to shell was estimated by dividing the dry weight minus the ash-free dry weight by the ash-free dry weight.

### Determination of the carbonate chemistry

The pH_NBS_ (mV) and temperature were measured in each jar prior to a water change using a potentiometric pH/temperature meter (Accumet Excel XL60). TRIS seawater buffer obtained from the Dickson Lab at Scripps Institution of Oceanography (SIO) was measured at three temperatures chosen to span the target temperatures in the experiment. The raw pH_NBS_ measurements (mV) were then converted to pH_T_ using the ideal pH calculation for TRIS at the nearest temperature to the sample [45]. One water sample from each treatment was collected for total alkalinity (TA) at each water change. In addition, water samples from all jars were collected on the same day for TA once a week. TA was measured using an automated Gran titration (Metrohm 809) standardized with a certified reference material from the Dickson Lab (SIO). Salinity was measured with a multi-parameter probe (YSI Professional Plus). Carbonate parameters were calculated from pH_T_, TA, and salinity as input in CO2SYS with K_1_ and K_2_ equilibrium constants from Merbach *et al.* (1973) and KHSO_4_ constants from Dickson (1990).

### Statistical analyses

For analyses of water chemistry and temperature, we calculated the mean values for each jar from the entirety of the experiment. These jar means were then used as individual replicates to test for differences among treatments using permutation-based ANOVA due to deviations from normality, with *temperature* and *CO_2_* as fixed factors, and *box* nested in *CO_2_*.

Variation in (1) the relative change in volume, (2) relative change in length, (3) the ratio of soft tissue mass to shell mass, and (4) the feeding rate was tested by fitting linear mixed effects models with *CO_2_* and *temperature* as fixed factors, and *box* nested in *CO_2_* using restricted maximum likelihood estimation in the *lme4* package in R [46]. The assumptions were checked visually, and Markov Chain Monte Carlo simulations were used to estimate p-values using the *pvals* function in R [47]. Using the *pvals* function, the intercept is that associated with the fitted model at 12°C and low CO_2_. Each contrast then compares how the addition of a particular factor alters the relationship relative to the one arising at 12°C and low CO_2_. Because the temperature effects are fit in ascending order (12 to 24°C), a significant effect of temperature at 16°C (p<0.05) for example, represents a significant difference at 16°C from the mean of the effects of temperatures at 12°C. The relative change in volume was also plotted against temperature for each CO_2_ treatment to compare differences in the shape of thermal performance curves with OA. Although different non-linear functions have been used to estimate thermal performance curves, we chose a quadratic function as the most simple, non-linear curve in order to discuss differences in shape. A separate quadratic curve was fit to each CO_2_ treatment in R.

Furthermore, we quantified the independent effect of the mean aragonite saturation state, *p*CO_2_, hydrogen ion (H^+^) concentration (as related to pH), and experimental temperature on the relative change in volume of mussels with hierarchical partitioning [48]. Hierarchical partitioning is a statistical technique that allows an estimation of the independent effects of highly collinear variables. This is done by calculating the average improvement in models (i.e., a goodness of fit measure) when each independent variable is incorporated across the hierarchy of models that use all combinations of independent variables. Hierarchical partitioning was performed with the hier.part package in R [49].

## Results

### Experimental conditions

The mean pH, aragonite saturation state, and *p*CO_2_ values differed significantly among CO_2_ and temperature treatments (Table 1 and S1). Within a CO_2_ treatment, the aragonite saturation state and pH were statistically lower in some of the coolest treatments than the warmest treatments. However, the differences were incremental and could not be distinguished statistically among all temperature treatments Table S1). Mean TA did not differ among temperature and/or CO_2_ treatments (Table 1). Mean temperature differed significantly among temperature treatments, but not between CO_2_ treatments of the same target temperature (*temperature*: *F*_5,20_ = 1559, p<0.001; Table 1). The mean standard deviation of the temperature in each jar was 0.9±0.4°C SD, primarily due to the cycling of the heaters. The mean standard deviation in seawater pH was 0.03±0.01 SD, probably due to respiration.

**Table 1:** Environmental and geochemical parameters in the jars.

Target CO_2_ (µatm)	Target temperature (°C)	Experimental temperature (°C)	pH_T_	TA (µmol/kgSW)	Ω Aragonite	*p*CO_2_ (µatm)
400	12	11.2±0.3	7.97±0.03	2401±199	2.07±0.1	545±42
	14	13.9±0.1	7.99±0.02	2366±188	2.24±0.1	516±51
	16	15.8±0.5	7.95±0.03	2376±190	2.33±0.2	536±32
	18	17.5±0.1	7.96±0.02	2367±169	2.43±0.2	539±25
	20	19.6±0.3	7.96±0.00	2356±160	2.54±0.1	553±56
	24	23.3±0.1	7.99±0.02	2441±218	3.13±0.2	497±30
1200	12	11.3±0.3	7.66±0.04	2384±194	1.06±0.1	1200±125
	14	13.7±0.4	7.67±0.04	2395±176	1.17±0.1	1169±74
	16	15.7±0.4	7.67±0.03	2381±177	1.29±0.1	1144±123
	18	17.9±0.3	7.65±0.04	2368±192	1.27±0.1	1251±145
	20	19.7±0.3	7.66±0.02	2378±205	1.39±0.0	1219±19
	24	23.3±0.3	7.70±0.04	2397±200	1.72±0.1	1102±137

Values represent the means of three jars ± SD. The target CO_2_ and temperature represent the assigned treatments levels. Experimental temperature, pH and TA were measured, while Ù Aragonite and *p*CO_2_ are calculated from pH and TA. Salinity was measured for water used to refill carboys and was the same for all treatments (although it differed among dates). Mean salinity was 33.8 ppt±0.2 (SD).

### Variation in mussel growth and feeding

**Figure 1:**
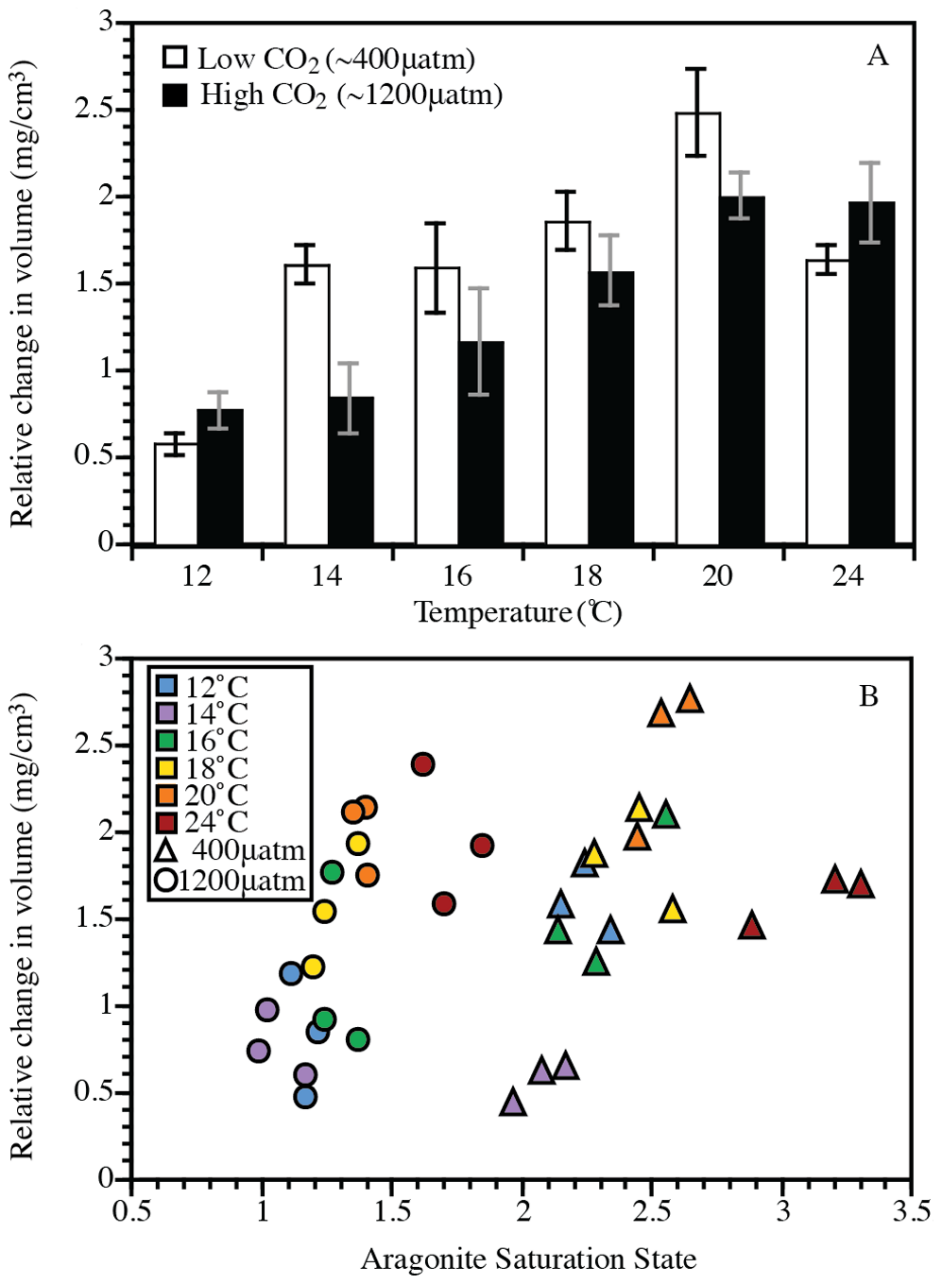
Change in volume vs. (A) treatment and (B) aragonite saturation state. (A) Mean relative change in volume of *Mytilus galloprovincialis* in two different CO_2_ treatments (400 µatm vs. 1200 µatm) and 6 temperature treatments (12, 14, 16, 18, 20, and 24°C). Mean is based on mean relative change in volume from each of three replicate jars (N = 3) at each treatment (± SEM). The mean change in volume of each jar is calculated from 5 *M. galloprovincialis* per jar. (B) Mean change in volume of *M. galloprovincialis* vs. the mean saturation state of aragonite in each jar.

At the coldest temperature (12°C), we did not detect a significant effect of CO_2_ on mussel growth (measured as a relative change in volume or length; Fig. 1A and Fig. S2). However, at 14°C, there was a significant interaction between temperature and CO_2_, with less growth in the high CO_2_ treatment. This trend continued with further warming (16–20°C), although the effect lessened with each subsequent 2°C rise in temperature such that we did not detect a statistically significant interaction between temperature and CO_2_ across the rest of the temperature range (i.e., 16–24°C; Table 2). In addition, the effect of reduced size in high CO_2_ was not at all apparent in the warmest temperature treatment (24°C). Because the results in the analyses of relative change in volume and relative change in length were similar, the length analyses are presented in Fig. S2 and Table S2.

**Table 2:** Mixed-effect model results for relative change in volume.

	Estimate	MCMC mean	HPD95 lower	HPD95 upper	*P* MCMC	
Intercept (12°C, 400 µatm)	0.5757	0.576	−0.5879	1.7408	0.2	
CO_2_ (1200 µatm)	0.1912	0.1949	−1.5094	1.7968	0.7	
Temperature (14°C)	1.0355	1.0313	0.4737	1.5856	0.002	**
Temperature (16°C)	1.0139	1.0099	0.4844	1.5724	<0.001	***
Temperature (18°C)	1.2814	1.2781	0.7158	1.8165	<0.001	***
Temperature (20°C)	1.9023	1.9014	1.3296	2.4392	<0.001	***
Temperature (24°C)	1.0539	1.0524	0.4946	1.5825	0.002	**
CO_2_ (1200)×Temp (14°C)	−0.9646	−0.9612	−1.7286	−0.1463	0.02	*
CO_2_ (1200)×Temp (16°C)	−0.6219	−0.6173	−1.3872	0.1611	0.1	
CO_2_ (1200)×Temp (18°C)	−0.481	−0.4781	−1.2506	0.3101	0.2	
CO_2_ (1200)×Temp (20°C)	−0.6692	−0.6665	−1.4841	0.0929	0.1	
CO_2_ (1200)×Temp (24°C)	0.1424	0.1448	−0.652	0.8845	0.7	

Output includes model estimates for fixed factors (Estimate) and the mean estimate across Markov chain Monte Carlo samples (MCMC mean), the upper and lower 95% highest posterior density intervals (HPD95 lower and HPD95 upper) and *p*-values based on the posterior distribution and Markov chain Monte Carlo sampling (*p* MCMC). Asterisks denote significance, with.

‘*’ = *P*<0.05,

‘**” = *P*<0.01, and

‘***” = *P*<0.001.

Because the mean aragonite saturation state increased with temperature in both low and high CO_2_ treatments (Table 1), the aragonite saturation state in the warmest high CO_2_ treatment (1200 µatm CO_2_, 24°C) and the coldest low CO_2_ treatment (400 µatm CO_2_, 12°C) were very similar (Fig. 1B). Despite this relative overlap in aragonite saturation states, the relative increase in volume of mussels at 12°C in the low CO_2_ treatment was 60% less than mussels in the 24°C high CO_2_ treatment. Hierarchical partitioning indicates that 67% of the variance in the relative change in volume of the mussels was explained independently by temperature, while 22% was explained independently by aragonite saturation state. The *p*CO_2_ and the concentration of the hydrogen ion explained relatively little of the variation in relative change in volume (6% and 5%, respectively).

**Figure 2:**
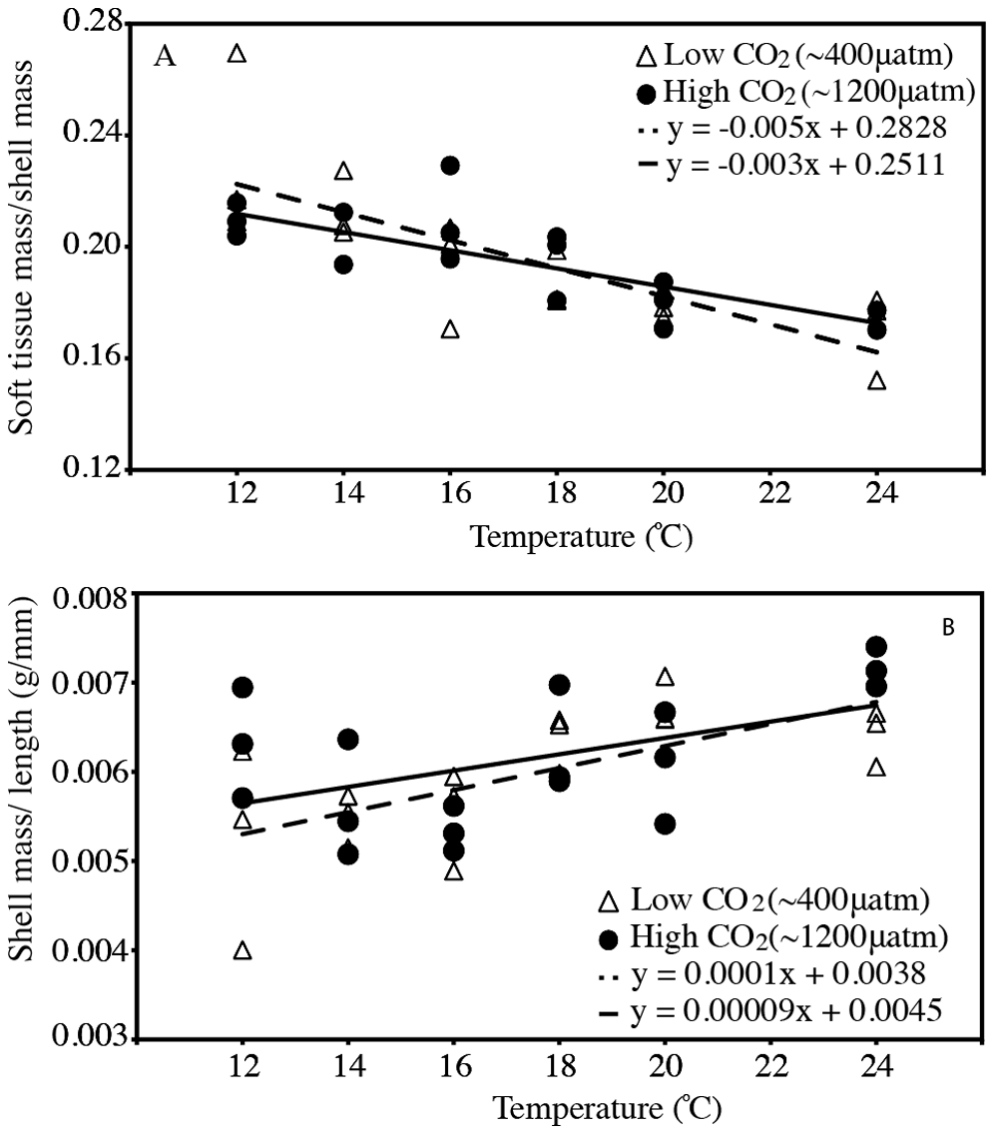
The relationship between temperature and the (A) ratio of soft tissue to shell mass and (B) length-corrected shell mass. Triangles = 400 µatm CO_2_, Circles = 1200 µatm CO_2_. Dotted line is the regression line fit to 400 µatm, while the solid line is the regression line fit to 1200 µatm. Data points represent the means of independent jars.

We did not detect consistent effects of CO_2_ on the relative ratio of soft tissue to shell mass. However, the ratio of soft tissue to shell mass consistently decreased with temperature (Table 3, Fig. 2A). This ratio was not dependent on the length of the mussels (*R^2^*_165_ = 0.01, *P* = 0.5), suggesting the temperature effect was not solely driven by higher growth rates in the warmer temperatures. Instead, the shell mass per total length increased with temperature (Fig. 2B *t* = 3.30, *P*_MCMC_ = 0.002), indicating that the mussels grew thicker shells in warmer treatments. In contrast, the soft tissue mass per total length did not differ among temperature treatments (*t* = −0.47, *P_MCMC_* = 0.6).

**Table 3:** Mixed-effect model results for ratio of soft tissue mass to shell mass.

	Estimate	MCMC mean	HPD95 lower	HPD95 upper	pMCMC	
Intercept (12°C, 400 µatm)	0.2318	0.2318	0.1816	0.2848	0.001	**
CO_2_ (1200 µatm)	−0.0222	−0.0223	−0.0912	0.0513	0.4	
Temperature (14°C)	−0.0184	−0.0184	−0.0441	0.006	0.1	
Temperature (16°C)	−0.0391	−0.0391	−0.0631	−0.0129	0.005	**
Temperature (18°C)	−0.0449	−0.0449	−0.0713	−0.0207	<0.001	***
Temperature (20°C)	−0.0526	−0.0527	−0.0778	−0.0275	<0.001	***
Temperature (24°C)	−0.0618	−0.062	−0.0874	−0.0374	<0.001	***
CO_2_ (1200)×Temp (14°C)	0.0087	0.0086	−0.0272	0.0449	0.6	
CO_2_ (1200)×Temp (16°C)	0.0395	0.0394	0.0032	0.0744	0.03	*
CO_2_ (1200)×Temp (18°C)	0.0303	0.0301	−0.0035	0.0638	0.9	
CO_2_ (1200)×Temp (20°C)	0.0226	0.0227	−0.0216	0.0585	0.2	
CO_2_ (1200)×Temp (24°C)	0.0248	0.025	−0.0112	0.0595	0.2	

Output is the same as in Table 2.

**Figure 3:**
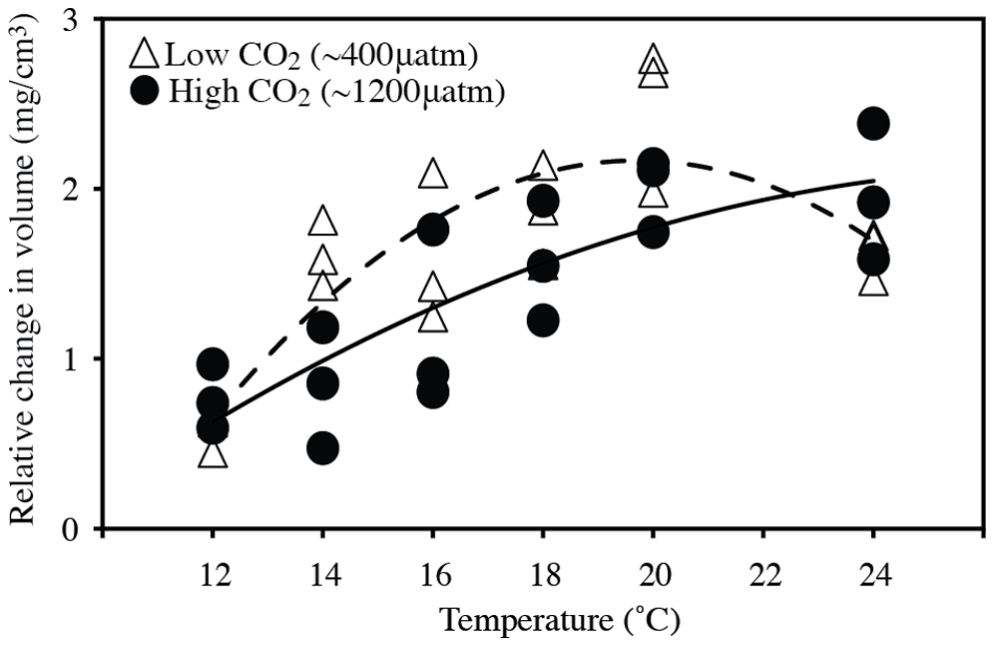
Thermal performance curves for each CO_2_ treatment. Curves are fit by a quadratic function. Triangles = 400 µatm CO_2_, Circles = 1200 µatm CO_2_. Dotted line is the curve fit to 400 µatm, while the solid line is the curve fit to 1200 µatm. Data points represent the means of independent jars.

The quadratic curves of change in volume vs. temperature reveal different responses among CO_2_ treatments. Growth increased with warmer temperatures from 12 to 20°C in both CO_2_ treatments (Fig. 3, Table 2). In the low CO_2_ treatment, the growth peaked at 20°C before declining at 24°C (y = 1.01x - 0.03x^2^ - 7.78, *R^2^* = 0.71, *P*<0.0001). In contrast, the growth was maintained at the highest temperature (24°C) in the high CO_2_ treatment (y = 0.343x - 0.006x^2^ - 2.589, *R^2^* = 0.68, *P* = 0.0002).

**Figure 4:**
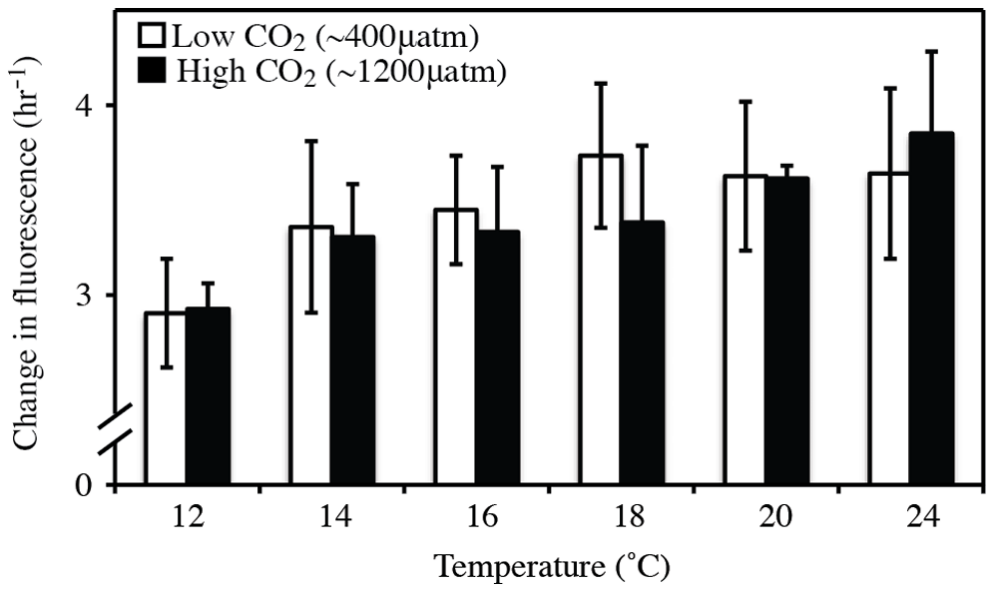
Variation in feeding rates among temperature and CO_2_ treatments. Mean change in fluorescence of microalgal mixture in seawater from two different CO_2_ treatments (400 µatm vs. 1200 µatm) and 6 temperature treatments (12, 14, 16, 18, 20, and 24°C) per hour. Mean is based on mean change in fluorescence from each of three replicate jars (N = 3) at each treatment (±SD) after allowing 20 hours for feeding.

Feeding rates (estimated by the change in fluorescence) increased with temperature from 12 to 18°C. Beyond 18°C, feeding rates were largely maintained at similar rates (Fig. 4). We did not detect a strong effect of CO_2_ on feeding rates (Table 4), although there were weak reductions in feeding rates in most high CO_2_ treatments compared to low CO_2_ treatments (Fig. 4).

**Table 4:** Mixed-effect model results for feeding rates.

	Estimate	MCMC mean	HPD95 lower	HPD95 upper	pMCMC	
Intercept (12°C, 400 µatm)	2.905	2.914	1.777	0.006	<0.001	***
CO_2_ (1200 µatm)	0.022	0.007	−1.581	0.973	0.9	
Temperature (14°C)	0.454	0.455	−0.059	0.087	0.06	
Temperature (16°C)	0.544	0.546	0.038	0.043	0.03	**
Temperature (18°C)	0.830	0.830	0.324	0.002	0.002	**
Temperature (20°C)	0.722	0.725	0.174	0.009	0.005	**
Temperature (24°C)	0.735	0.736	0.203	0.007	0.005	**
CO_2_ (1200)×Temp (14°C)	−0.075	−0.075	−0.813	0.836	0.8	
CO_2_ (1200)×Temp (16°C)	−0.139	−0.138	−0.882	0.706	0.7	
CO_2_ (1200)×Temp (18°C)	−0.374	−0.378	−1.093	0.306	0.3	
CO_2_ (1200)×Temp (20°C)	−0.035	−0.036	−0.744	0.924	0.9	
CO_2_ (1200)×Temp (24°C)	0.189	0.188	−0.580	0.598	0.6	

Output is the same as in Table 2.

## Discussion

Our results indicate that the biological effects of OA on the growth of bay mussels are highly dependent on temperature. The effects of OA were most apparent at 14°C, a temperature commonly experienced by this population in the field. The reduction in growth associated with elevated CO_2_ at 14°C, however, lessened with each subsequent 2°C step warming up to 20°C, suggesting that warmer temperatures can offset some of the potentially detrimental effects of OA on mussel growth. In addition, the lack of significant CO_2_ effects on mussel growth at the coldest (12°C) and warmest (24°C) temperatures indicates that temperature was the key factor in our study driving growth at the most extreme temperatures in our experiment.

While it is well understood that temperature can directly affect organisms' physiology, growth, and behavior [14], [15], [50], the indirect effects of temperature on marine organisms due to its effects on carbonate chemistry (i.e., increased aragonite saturation states and pH in warmer treatments) have garnered less attention (but see [51], [52]). Because these two mechanisms are acting simultaneously, it is difficult to attribute the interactive effects of temperature and CO_2_ to one or the other. The relationship between aragonite saturation state and mussel growth (Fig. 1B), however, provides insight into the direct versus indirect effects of temperature on the mussels' response to OA. Because the aragonite saturation states were very similar between the warmest (24°C) high CO_2_ treatments and the coldest (12°C) low CO_2_ treatments (Fig. 1B), we can rule out the indirect effect of temperature via increased aragonite saturation states between these two specific treatments. Instead, the 60% difference in growth between these two treatments can be attributed primarily to the direct effects of temperature on mussel physiology since their aragonite saturation states were so similar. Furthermore, as noted above, the results from the hierarchical partitioning indicate that temperature independently explains 67% of the differences in growth, while aragonite saturation state explains significantly less (∼22%). These results suggest that the amelioration of OA impacts with rising temperature is most likely due to the direct effects of temperature on mussel physiology and highlight the disproportionate influence of temperature on mussel shell growth.

Although early studies of *M. galloprovincialis* indicated that reduced growth associated with extremely low pH (∼7.3) was likely due to metabolic depression [37], studies of other mussel and bivalve species have concluded that reduced growth associated with more moderate pH reductions is due to increased energetic demand for homeostatic processes [53], [54]. Mussels do not compensate for extracellular pH changes caused by OA [37], [55], including the pH changes in the extrapallial fluid that surrounds the inner surface of the shell. Thus, the maintenance of the inner shell surfaces and prevention of dissolution in lower pH/higher *p*CO_2_ conditions requires more energy than in low CO_2_ [24]. Coupled with the increased energetic demand for homeostasis in high CO_2_ conditions, altered allocation to maintenance could limit the energy available for shell growth [24].

We hypothesize that the reduced effect of OA with warming may be due to increased energy supply at warmer temperatures. For *M. galloprovincialis*, moderate warming can increase the amount of time that the valves are open [56], which would allow more time for active feeding. Furthermore, water viscosity decreases and the rate of pumping increases with temperature, potentially allowing for more rapid feeding in warmer temperatures [26], [57], [58]. Our results indicate that feeding rates increased with temperature up to 18°C, after which they were maintained at 20–24°C. This may be due to mussels spending more time with their valves closed once water temperatures become stressful [33], [56], [59]. A previous long-term study of *M. galloprovincialis* found that substantial valve closure under constant immersion occurred at approximately 17–20°C [56]. Although we did not measure valve closure in our experiment, this research is consistent with our results and suggests that warming could lead to higher feeding rates and enhanced scope for growth of immersed mussels at temperatures below 20°C, which could offset the energetically costly process of shell maintenance and homeostasis associated with OA. While the present study examined the effects of temperature and OA under constant immersion, it should be noted that valve closure responses differ in mussels exposed to warming in air and seawater [59]. In particular, valve closure in air tends to last longer and occur at lower temperatures [59]. Thus, our results are most applicable to mussels exposed to constant immersion, such as those living on docks and in aquaculture facilities, and care should be employed when considering intertidal mussels exposed to daily emersion regimes. Regardless, our results suggest that energy budgets could play an important role in the interaction between temperature and OA.

It should also be noted that the pH values of intracellular fluids vary regularly with temperature, such that intracellular pH of mussels is expected to be approximately 0.2 units lower in the warmest treatment (24°C) than the coolest treatment of our experiment (12°C) [14]. Intracellular pH is normally maintained below that of the surrounding seawater, and the correspondent high intracellular *p*CO_2_ drives diffusive transport of metabolic CO_2_ out of cells. In the low CO_2_ treatment (400 µatm), the reduction in intracellular pH caused by warming would likely increase the gradient between intracellular pH/*p*CO_2_ and the surrounding seawater, which could increase diffusion of CO_2_ out of cells. However at high CO_2_ (1200 µatm), the intracellular pH in the coolest treatment (12°C) would be more alkaline than seawater, while the intracellular pH in the warmest treatment (24°C) would likely be very similar to seawater pH [14]. Thus, the gradient between seawater pH and intracellular pH likely varies among temperatures and CO_2_ treatments, which may influence the energetics and calcification rates in non-intuitive ways [60], [61]. Further research is needed to understand how these factors influence species' responses to OA.

While our results indicate that the direct effects of temperature play a fundamental role in the mussels' responses to CO_2_, we cannot rule out the indirect effects of temperature completely. Instead, we suggest that the relative importance of the direct and indirect effects may differ with the environmental conditions. Some physiological models of multiple stressors predict that OA will contract the organism's thermal tolerance window by limiting the capacity for oxygen consumption [62], [63], however our results highlight a potential broadening of the thermal tolerance peak in the higher CO_2_ treatment based on the differences in shape of the quadratic curves (Fig. 3). Further experiments are needed that test mussel performance beyond the temperatures used here to better understand these trends and their robustness, but our results reveal an interesting pattern. We hypothesize that this pattern may be due in part to the indirect effects of temperature on saturation state, which is not included in these physiological models. While the direct temperature effects at 24°C might include a reduction in feeding and growth regardless of CO_2_ concentration, we hypothesize that the increase in temperature from 20 to 24°C in the high CO_2_ treatment may also indirectly benefit the mussels by increasing the saturation state. This temperature-mediated increase in saturation state is also happening in the low CO_2_ treatment, but it is likely that there are threshold values in saturation state beyond which increases in saturation state have very little effect on growth or calcification [64] and this mechanism would be less important.

Our results also reveal that the mussels grew thicker shells in the warmer conditions regardless of CO_2_ levels. Because the rate of non-biogenic calcium carbonate precipitation increases with temperature, the thicker shells in warmer conditions could signal less costly calcification in the warmer treatments [29], [51]. However, the mechanisms of biogenic calcification are still poorly understood, and this effect could also signal that the mussels in our experiment allocated the excess energy available in warmer temperatures towards the production of a thicker shell. This could in part be explained by higher feeding rates or the increased availability of mitochondrial CO_2_ associated with respiration that can be converted into HCO_3_^−^ for calcification in warmer treatments [65], [66]. Moreover, a thicker shell could potentially protect juvenile mussels from predation. This effect appears to be independent of CO_2_ levels, which could be because the effects of OA were modest across most of the range of temperatures tested.

Although elevated temperature has been shown to exacerbate the effects of ocean acidification in a wide range of experiments [11], moderate warming has been shown to offset some of the detrimental effects of OA in several species. For example, Chan *et al.* (2013) found that elevated temperature offset the negative effects of reduced pH and salinity on the accretion and strength of calcareous tubes of tropical tubeworms [67]. Similarly, moderate warming was found to slightly ameliorate the negative effects of OA on the shell growth of larval oysters (although more extreme warming exacerbated the effects of OA) [68]. These results could in part be explained by the potential for warming mediated effects on geochemistry to benefit processes related with calcification or shell growth. A better understanding of whether the direct or indirect effects of temperature are driving the interactions may lend some insight into in the conditions under which warming ameliorates or exacerbates the effects of OA.

In conclusion, our results demonstrate that the effects of OA are highly dependent on environmental temperatures, and that warmer temperatures can mediate some of the effects of OA on mussels via both direct physiological and indirect geochemical pathways. The combined effects of OA and temperature highlighted here are likely to be mediated by other factors, including food availability [24], [25], [27], carry-over effects [39], [69], [70], and environmental variability [71], [72]. Further attention to these other factors is necessary to better understand the ecological significance of our findings. Based on the interactions examined here, however, our results suggest that small *M. galloprovincialis* mussels may be most vulnerable to OA/high CO_2_ in cooler temperatures, which will vary seasonally and geographically. In the absence of acclimatization or local adaptation [57], [73], our results suggest the effects of OA could be most pronounced at the polar edges of *M. galloprovincialis'* range and in upwelling dominated environments where high CO_2_ and cold temperatures are tightly linked. Importantly, our results suggest that *M. galloprovincialis* may be able to tolerate increasing *p*CO_2_ and decreasing aragonite saturation state across a wide range of temperatures they naturally experience in the field, with moderate warming offsetting potential reductions in growth associated with OA.

Beyond this model species, our results highlight the importance of considering multiple ecologically relevant parameters in global change experiments. Species currently exist across a complex range of conditions, and the routine environmental variability experienced by species often exceeds the magnitude of changes projected with global climate change. Therefore, refining our forecasts of the ecological effects of global change requires a mechanistic understanding of the interactive effects of multiple environmental drivers across the full suite of conditions experienced now as well as those projected to occur in coming decades.

## Supporting Information

Figure S1 Schematic of experimental setup. (A) Each Plexiglas box contained six experimental jars. Pre-mixed gas was pumped into the headspace of each box, which was then bubbled into the jars, such that all jars in the same box were at the same CO_2_ level. (B) Each jar was heated individually using submersible heaters. (C) Temperature treatments were haphazardly assigned to each box.
(TIF)

Figure S2 Variation in change in length among treatments. Mean relative change in length of *Mytilus galloprovincialis* in two different CO_2_ treatments (400 µatm vs. 1200 µatm) and 6 temperature treatments (12, 14, 16, 18, 20, and 24°C). Mean is based on mean relative change in length from each of three replicate jars (N = 3) at each treatment (±SEM). The mean change in length of each jar is calculated from 5 *M. galloprovincialis* per jar.
(TIF)

Table S1 *F*-statistics and p-values from two-way crossed ANOVAs of major carbonate chemistry parameters, with temperature and CO_2_ as fixed factors. *F*-statistics are not reported for non-significant *p*-values.
(DOCX)

Table S2 Mixed-effect model results for relative change in length. Output includes model estimates for fixed factors (Estimate) and the mean estimate across Markov chain Monte Carlo samples (MCMC mean), the upper and lower 95% highest posterior density intervals (HPD95 lower and HPD95 upper) and *p*-values based on the posterior distribution and Markov chain Monte Carlo sampling (*p* MCMC). Asterisks denote significance, with ‘*’ = *P*<0.05, ‘**” = *P*<0.01, and ‘***” = *P*<0.001.
(DOCX)
