# Supplementary figures and images for: The Role of Temperature in Determining Species' Vulnerability to Ocean Acidification: A Case Study Using Mytilus galloprovincialis
Source: PLoS One. 2014 Jul 1;9(7):e100353. doi: 10.1371/journal.pone.0100353 (PMC4077567; doi:10.1371/journal.pone.0100353)

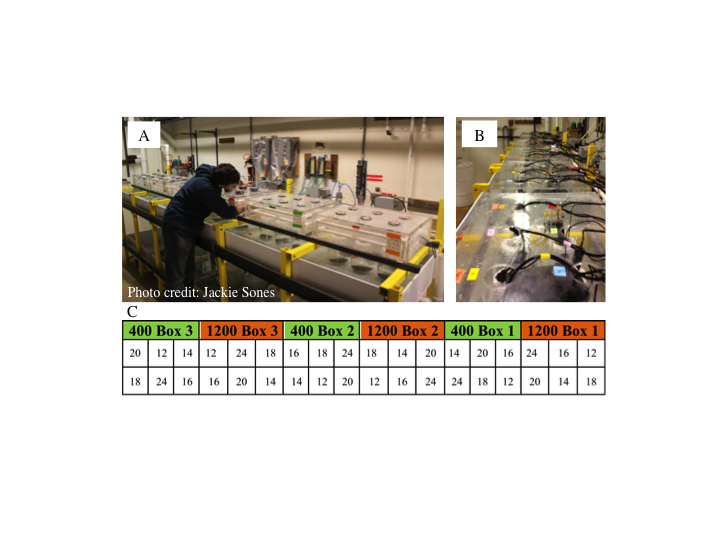

Supplement: Figure S1 — Schematic of experimental setup. (A) Each Plexiglas box contained six experimental jars. Pre-mixed gas was pumped into the headspace of each box, which was then bubbled into the jars, such that all jars in the same box were at the same CO2 level. (B) Each jar was heated individually using submersible heaters. (C) Temperature treatments were haphazardly assigned to each box. (TIF) [file pone.0100353.s001.tif]

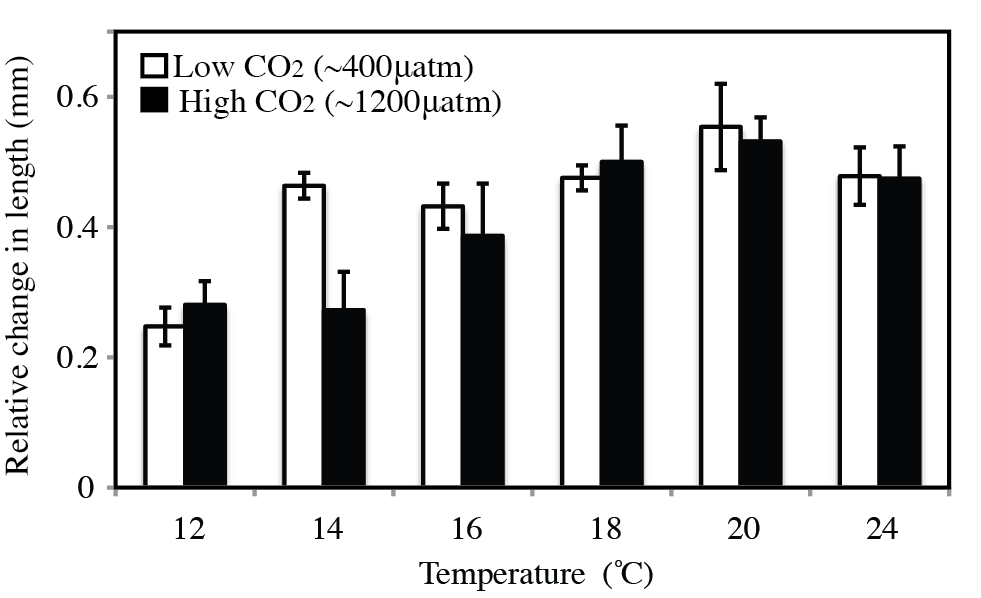

Supplement: Figure S2 — Variation in change in length among treatments. Mean relative change in length of Mytilus galloprovincialis in two different CO2 treatments (400 µatm vs. 1200 µatm) and 6 temperature treatments (12, 14, 16, 18, 20, and 24°C). Mean is based on mean relative change in length from each of three replicate jars (N = 3) at each treatment (±SEM). The mean change in length of each jar is calculated from 5 M. galloprovincialis per jar. (TIF) [file pone.0100353.s002.tif]
