# Supplementary material for: The Role of Temperature in Determining Species' Vulnerability to Ocean Acidification: A Case Study Using Mytilus galloprovincialis
Source: PLoS One. 2014 Jul 1;9(7):e100353. doi: 10.1371/journal.pone.0100353 (PMC4077567; doi:10.1371/journal.pone.0100353)
Supplement: Table S1 — F-statistics and p-values from two-way crossed ANOVAs of major carbonate chemistry parameters, with temperature and CO2 as fixed factors. F-statistics are not reported for non-significant p-values. (DOCX) [file pone.0100353.s003.docx]

| Factors | Temperature | CO_2_ | Temperature x CO_2_ |
| --- | --- | --- | --- |
| Temperature | F_5,35_ = 1559, *p* = 0.0001 | *p* = 0.8 | *p* = 0.4 |
| pH_T_ | F_5,35_ = 7, *p* <0.001 | F_1,35_ = 184, *p*<0.001 | *p* = 0.4 |
| TA | p = 0.99 | *p* = 0.99 | *p* = 0.99 |
| Aragonite | F_5,35_ = 90, *p* = 0.0001 | F_1,35_ = 188, *p*<0.001 | F_5,35_ = 5, *p* = 0.002 |
| *p*CO_2_ | F_5,35_ = 4, *p* = 0.02 | F_1,35_ = 120, *p*<0.001 | *p* = 0.3 |
