# Supplementary material for: The Role of Temperature in Determining Species' Vulnerability to Ocean Acidification: A Case Study Using Mytilus galloprovincialis
Source: PLoS One. 2014 Jul 1;9(7):e100353. doi: 10.1371/journal.pone.0100353 (PMC4077567; doi:10.1371/journal.pone.0100353)
Supplement: Table S2 — Mixed-effect model results for relative change in length. Output includes model estimates for fixed factors (Estimate) and the mean estimate across Markov chain Monte Carlo samples (MCMC mean), the upper and lower 95% highest posterior density intervals (HPD95 lower and HPD95 upper) and p-values based on the posterior distribution and Markov chain Monte Carlo sampling (p MCMC). Asterisks denote significance, with ‘*’ = P<0.05, ‘**” = P<0.01, and ‘***” = P<0.001. (DOCX) [file pone.0100353.s004.docx]

| Length ~ CO_2_*Temp + (1\|CO_2_/box) | | |  |  |  |  |
| --- | --- | --- | --- | --- | --- | --- |
|  |  |  |  |  |  |  |
|  | | | | | |  |
|  | Estimate | MCMC mean | HPD95 lower | HPD95 upper | pMCMC |  |
| Intercept (12ºC, 400 µatm) | 0.2475 | 0.2465 | -0.0581 | 0.692 | <0.001 | *** |
| CO_2_ (1200 µatm) | 0.0332 | 0.0344 | -0.3856 | 0.7588 | 0.7 |  |
| Temperature (14ºC) | 0.2159 | 0.2148 | 0.0773 | 0.005 | 0.004 | ** |
| Temperature (16ºC) | 0.1844 | 0.1835 | 0.051 | 0.0084 | 0.01 | ** |
| Temperature (18ºC) | 0.228 | 0.2272 | 0.0873 | 0.0016 | 0.002 | ** |
| Temperature (20ºC) | 0.3062 | 0.306 | 0.1643 | 0.0001 | <0.001 | *** |
| Temperature (24ºC) | 0.2307 | 0.2303 | 0.0918 | 0.001 | 0.002 | ** |
| CO_2_ (1200) x Temp (14ºC) | -0.2239 | -0.2231 | -0.4113 | 0.0278 | 0.02 | * |
| CO_2_ (1200) x Temp (16ºC) | -0.0784 | -0.0773 | -0.2657 | 0.4148 | 0.4 |  |
| CO_2_ (1200) x Temp (18ºC) | -0.0085 | -0.0078 | -0.2055 | 0.9378 | 0.9 |  |
| CO_2_ (1200) x Temp (20ºC) | -0.0555 | -0.0548 | -0.2527 | 0.564 | 0.6 |  |
| CO_2_ (1200) x Temp (24ºC) | -0.0369 | -0.0363 | -0.2313 | 0.7024 | 0.7 |  |
